# Implementation of Telehealth Services at the US Department of Veterans Affairs During the COVID-19 Pandemic: Mixed Methods Study

**DOI:** 10.2196/29429

**Published:** 2021-09-23

**Authors:** Claudia Der-Martirosian, Tamar Wyte-Lake, Michelle Balut, Karen Chu, Leonie Heyworth, Lucinda Leung, Boback Ziaeian, Sarah Tubbesing, Rashmi Mullur, Aram Dobalian

**Affiliations:** 1 Veterans Emergency Management Evaluation Center US Department of Veterans Affairs North Hills, CA United States; 2 Center for the Study of Healthcare Innovation, Implementation & Policy Veterans Affairs Greater Los Angeles Healthcare System Los Angeles, CA United States; 3 Office of Connected Care/Telehealth Services Veterans Health Administration Washington, DC United States; 4 Department of Medicine University of California San Diego School of Medicine San Diego, CA United States; 5 Division of General Internal Medicine-Health Services Research David Geffen School of Medicine University of California Los Angeles Los Angeles, CA United States; 6 Division of Cardiology David Geffen School of Medicine University of California Los Angeles Los Angeles, CA United States; 7 Department of Medicine David Geffen School of Medicine University of California Los Angeles Los Angeles, CA United States; 8 Division of Diabetes, Endocrinology & Metabolism David Geffen School of Medicine University of California Los Angeles Los Angeles, CA United States; 9 Division of Health Systems Management and Policy School of Public Health University of Memphis Memphis, TN United States

**Keywords:** telehealth, telemedicine, veterans, US Department of Veterans Affairs, primary care, cardiology, home-based primary care, COVID-19

## Abstract

**Background:**

At the onset of the COVID-19 pandemic, there was a rapid increase in the use of telehealth services at the US Department of Veterans Affairs (VA), which was accelerated by state and local policies mandating stay-at-home orders and restricting nonurgent in-person appointments. Even though the VA was an early adopter of telehealth in the late 1990s, the vast majority of VA outpatient care continued to be face-to-face visits through February 2020.

**Objective:**

We compared telehealth service use at a VA Medical Center, Greater Los Angeles across 3 clinics (primary care [PC], cardiology, and home-based primary care [HBPC]) 12 months before and 12 months after the onset of COVID-19 (March 2020).

**Methods:**

We used a parallel mixed methods approach including simultaneous quantitative and qualitative approaches. The distribution of monthly outpatient and telehealth visits, as well as telephone and VA Video Connect encounters were examined for each clinic. Semistructured telephone interviews were conducted with 34 staff involved in telehealth services within PC, cardiology, and HBPC during COVID-19. All audiotaped interviews were transcribed and analyzed by identifying key themes.

**Results:**

Prior to COVID-19, telehealth use was minimal at all 3 clinics, but at the onset of COVID-19, telehealth use increased substantially at all 3 clinics. Telephone was the main modality of patient choice. Compared with PC and cardiology, video-based care had the greatest increase in HBPC. Several important barriers (multiple steps for videoconferencing, creation of new scheduling grids, and limited access to the internet and internet-connected devices) and facilitators (flexibility in using different video-capable platforms, technical support for patients, identification of staff telehealth champions, and development of workflows to help incorporate telehealth into treatment plans) were noted.

**Conclusions:**

Technological issues must be addressed at the forefront of telehealth evolution to achieve access for all patient populations with different socioeconomic backgrounds, living situations and locations, and health conditions. The unprecedented expansion of telehealth during COVID-19 provides opportunities to create lasting telehealth solutions to improve access to care beyond the pandemic.

## Introduction

At the onset of the COVID-19 pandemic, there was a rapid increase in the use of telehealth services at the US Department of Veterans Affairs (VA) [1,2], which was accelerated by state and local policies mandating stay-at-home orders and restricting nonurgent in-person appointments. Even though the VA was an early adopter of telehealth in the late 1990s, the vast majority of VA outpatient care continued to be face-to-face visits through February 2020 [1]. To provide safe and effective access to care amid the COVID-19 pandemic, many VA sites and health care providers across the nation switched from conventional face-to-face outpatient visits to virtual encounters practically overnight.

Previous VA and non-VA telehealth studies have examined telehealth use and outcomes in situations where patients and clinicians had a choice between virtual and in-person services [3-10]. With the onset of COVID-19, however, the use of telehealth quickly became a necessity rather than a choice. This rapid expansion of various modalities at VA sites across the nation has provided new opportunities for research both within and outside of the VA [2,11-18]. Currently, there is a gap in the literature regarding telehealth adoption and implementation during the COVID-19 era, especially across various specialty clinics.

VA medical centers (VAMCs) house a variety of clinics, which can all vary in their structures and processes. This study focused on the use of telehealth services at 3 distinct clinics (primary care [PC], cardiology, and home-based primary care [HBPC]) at a VAMC, VA Greater Los Angeles, California (GLA), and associated community-based outpatient clinics (CBOCs). PC is a gateway to all other care types in the VA, and veterans rely on it for the management of both acute and chronic conditions. Cardiology manages highly acute and medically complex patients, who might be at high risk for hospitalization. HBPC has both a highly vulnerable population and a unique framework for supporting patients in their homes [19,20].

The main objective of this study was to compare the use and rapid uptake of telehealth services in a health care system across 3 clinics (PC, cardiology, and HBPC) 12 months before and 12 months after the onset of COVID-19. The quantitative analysis provides an overview of the expansion of virtual care services at each clinic during the 1-year COVID-19 period. The qualitative analysis illustrates the barriers and facilitators to achieving rapid implementation of telehealth services during and immediately after the onset of COVID-19 across the 3 clinics.

## Methods

For this study, a parallel mixed methods approach was used where quantitative data management/analyses and qualitative data collection/analyses were conducted simultaneously. For the *quantitative* portion, VA administrative and clinical data from the VA Corporate Data Warehouse were used. Outpatient visits were identified as either telehealth or nontelehealth in-person encounters. Even though telephone care at the VA is not considered synchronous telemedicine according to national guidance, since the VA allows for telephone and telehealth care to be reimbursed at the same rate as face-to-face care during COVID-19, telehealth is defined for the purposes of this study as direct patient care over a distance, regardless of what type of modality is used [21], telephone or video. Asynchronous telehealth and remote patient monitoring were not included in this definition of telehealth.

Based on input from the project’s clinical coinvestigators, guidance from the telemedicine outpatient protocols, and previously published work, “telehealth” and “in-person” visits were identified by filtering the patient encounter data on clinic codes, location names (tele vs nontele visit), and current procedural terminology (CPT) codes. Clinic codes are 3-digit numeric identifiers that correspond to the work group primarily responsible for providing a clinical service during an outpatient encounter. A CPT code is a 5-character numeric or alphanumeric code that is assigned to every task and service provided to a patient during an encounter, some of which correspond to telehealth services. Location names represent geographic location and clinic grid names, which help to determine whether it is a telehealth visit or in-person visit.

For each clinic/program (PC, cardiology, and HBPC), a distinct study cohort was identified. Veterans were included in a clinic cohort if they had at least one visit to the clinic 1 year prior to March 1, 2020. The PC study cohort included 64,361 patients (299,881 visits) 12 months before COVID-19 and 48,729 patients (247,849 visits) 12 months after the onset of COVID-19. The corresponding numbers for cardiology were 5527 patients (14,229 visits) and 3690 patients (10,800 visits), and for HBPC were 240 patients (4102 visits) and 162 patients (3929 visits) (Multimedia Appendix 1).

For the analysis, the total number of monthly outpatient and telehealth visits 12 months before (March 1, 2019, through February 28, 2020) and 12 months after the onset of COVID (March 1, 2020, through March 1, 2021) were calculated for each clinic (PC, cardiology, and HBPC). For this study, VA Video Connect (VVC) includes a videoconferencing app approved by the VA that helps connect veterans with their health care providers via a secure and private session, as well as other non-VVC video technologies such as Doximity and FaceTime.

For the *qualitative* portion, semistructured 30-minute telephone interviews were conducted with 34 GLA staff members who were involved in providing or supporting telehealth services within PC, cardiology, and HBPC during the COVID-19 pandemic. Respondents included 18 clinical providers (physicians, nurse practitioners, registered nurse care managers, and clinical fellows), 8 ancillary providers (social workers, psychologists, dieticians, pharmacists, and occupational therapists), 5 nurse managers, and 3 Health Administration Service leaders.

All telephone interviews were conducted by two to three members of the research team from July to October 2020. The interview guide, which was developed with guidance from the clinical coinvestigators queried respondents about (1) facility and clinic preparedness policies and procedures on the transition to telehealth; (2) types of support received when transitioning to telehealth; (3) how telehealth appointments were scheduled, tracked, and coded; (4) types of modalities of telehealth delivery used; and (5) types of facilitators and barriers experienced during telehealth implementation.

All interviews were audio recorded and transcribed. The study team utilized a rapid analysis approach, which produces effective, contextually rich, valid, and timely results [22,23] to analyze the interview transcripts and prepare the dissemination of findings. The first analytic step involved developing a templated summary table of key domains based on the interview guide. The draft summary table was reviewed and modified after being tested by the analytic team with a single transcript. Using the updated templated summary table, which reflected additional domains that emerged from the data in the initial collective analysis, all transcripts were divided and independently summarized by the study team members. Then, each team member conducted a randomized secondary review of five to six summaries and discussed discrepancies with the team to ensure consistency in the data being recorded. The second analytic step involved consolidating the summaries into 3 high-level summary documents (1 for each clinic) to identify key points and commonly occurring themes across all interviews. Clinical coinvestigators, who represented lead positions from each of the 3 clinics, discussed and confirmed the identified themes and their value to future telehealth implementation efforts. This study was approved by the VA GLA Institutional Review Board.

## Results

### Overview

There was a shift in outpatient services, where the volume of all outpatient visits after the onset of COVID-19 decreased for all 3 clinics (PC, −17.4%; cardiology, −24.1%; HBPC, −4.2%). In terms of unique patients, the number of patients who accessed outpatient services at all 3 clinics 12 months before compared to 12 months after the onset of COVID-19 also decreased (PC, −24.3%; cardiology, −33.2%; HBPC, −32.5%; Multimedia Appendix 1).

**Figure 1:**
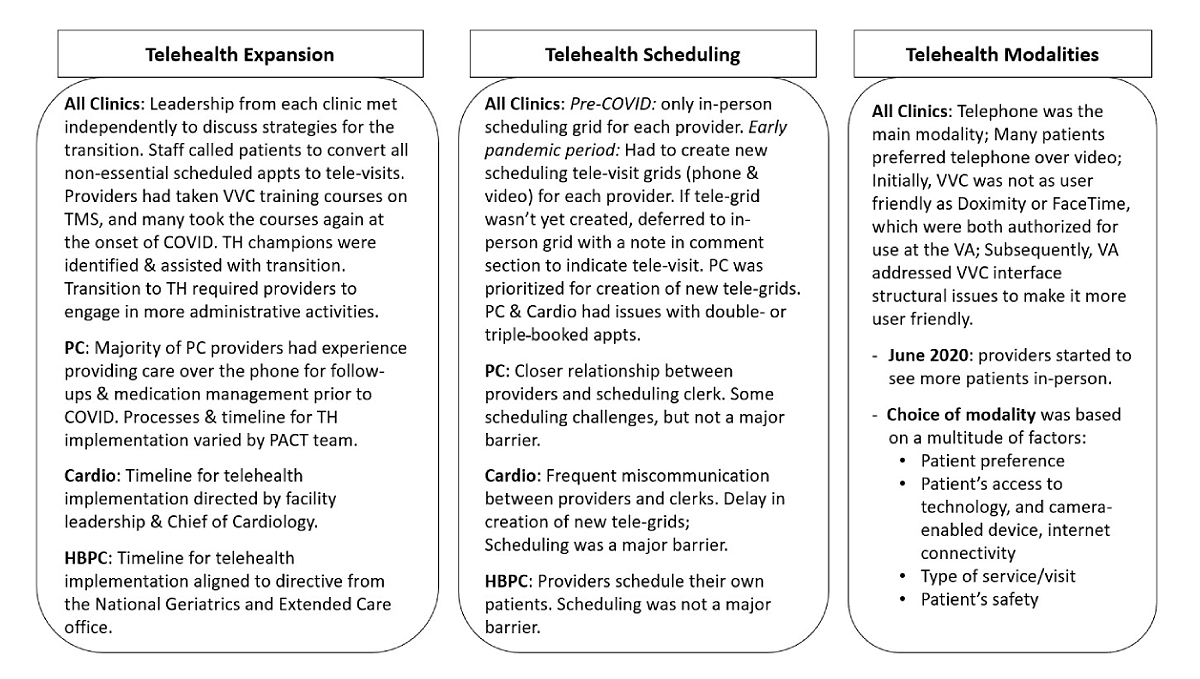
Implementation of telehealth at Veterans Affairs Greater Los Angeles, California during COVID-19. Appts: appointments; HBPC: home-based primary care; PACT: patient aligned care team; PC: primary care; TH: telehealth; TMS: talent management system; VA: US Department of Veterans Affairs; VVC: US Department of Veterans Affairs Video Connect.

Figure 1 displays the summary from the qualitative analysis. The following 3 main themes emerged regarding the transition to telehealth services: (1) telehealth expansion, (2) telehealth scheduling, and (3) telehealth modalities. Within each of these themes, respondents identified key barriers and facilitators to the rapid implementation of telehealth.

### Telehealth Expansion

**Figure 2:**
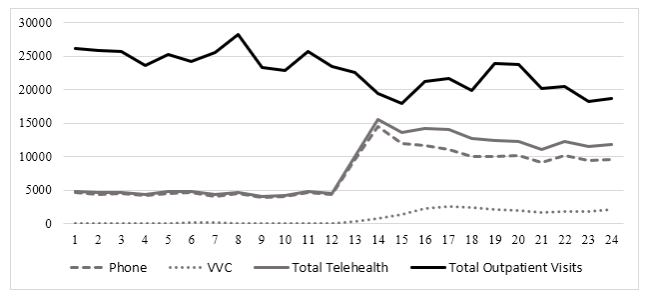
Total number of outpatient encounters in primary care at Veterans Affairs Greater Los Angeles, California (March 1, 2019, through March 1, 2021) by the care delivery method. VVC: US Department of Veterans Affairs Video Connect.

**Figure 3:**
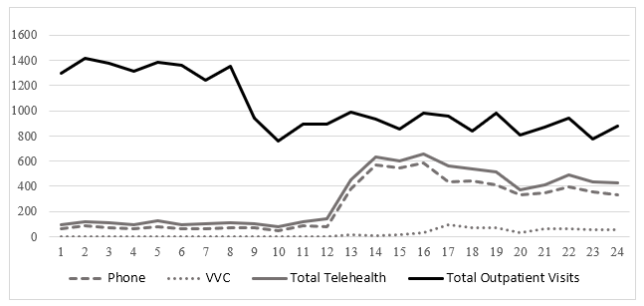
Total number of outpatient encounters in cardiology at Veterans Affairs Greater Los Angeles, California (March 1, 2019, through March 1, 2021) by the care delivery method. VVC: US Department of Veterans Affairs Video Connect.

**Figure 4:**
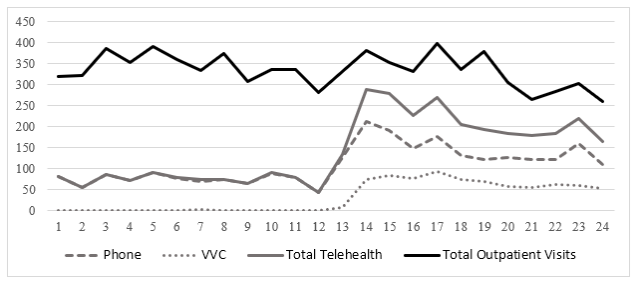
Total number of outpatient visits in home-based primary care at Veterans Affairs Greater Los Angeles, California (March 1, 2019, through March 1, 2021) by the care delivery method. VVC: US Department of Veterans Affairs Video Connect.

Figures 2-4 illustrate the total number of monthly outpatient and telehealth encounters for PC, cardiology, and HBPC 12 months before (March 1, 2019, through February 28, 2020) and 12 months after the onset of COVID-19 (March 1, 2020, through March 1, 2021) at GLA. The findings indicate that before the onset of COVID-19, for all 3 clinics, telehealth use varied between 4116 and 4849 for PC (Figure 2), 77 and 139 for cardiology (Figure 3), and 44 and 91 for HBPC (Figure 4). At the onset of COVID (during March 2020), telehealth use increased substantially after the onset of COVID-19 and reached its peak at 15,480 for PC in May 2020. For cardiology and HBPC, the peak was 654 telehealth visits (July 2020) and 289 telehealth visits (May 2020), respectively. Starting in August 2020, the use of telehealth services for all 3 clinics started to decline slightly, but never reached pre–COVID-19 levels during the 12 months after the onset of COVID-19 (Figures 2-4).

According to our qualitative findings, all study respondents indicated that the rapid transition to telehealth was driven by the dual declarations of the VAMC suspending all nonurgent procedures on March 17, 2020, and the Governor of California declaring a state of emergency and issuing a stay home order on March 19, 2020. VA leadership at all 3 major levels (the local medical facility level, regional Veterans Integrated Service Networks, and VA National Office) pushed for the rapid switch to telehealth. Even though all clinics were instructed to mirror their face-to-face grids with telehealth services, there were issues on how to actualize the implementation, so the processes of transitioning patients from face-to-face visits to virtual visits varied by clinic. Leadership from each clinic met independently to discuss strategies for the transition. As an example, the processes and timeline of the transition to telehealth varied by the PC team. One member commented as follows:

I remember talking to my colleagues about are we overreacting, should we be moving to in-person? …everything was coming out in the news and we weren’t really sure how big of a deal this was. So, at least for a couple of weeks, we were sort of making the determination on our own, should we just be proactively calling all of our patients and telling them we’re doing telehealth? … Should we just see people in-person, that this [pandemic] isn’t that big of a deal? So, at least for a couple of weeks, that was the way it worked.

PC respondent #201

The HBPC program received direction from the National Geriatrics and Extended Care office to limit face-to-face care to only essential visits, and on March 16, 2020, the HBPC program Director sent an email instructing staff to do all nonessential visits over the phone or over video. Lastly, a leader in cardiology acted as a champion to quickly convert the department to virtual care and commented as follows:

I was one of the earlier alarmists about the virus and the pandemic… We just internally decided to implement our own policy within our division and then everyone had buy-in pretty much on our faculty meeting. So, I think in the beginning, I instigated, like this is what we need to do now and how are we going to do it.

Cardiology respondent #101

Once the decision was made to transition to telehealth, staff in all 3 clinics quickly began calling all patients to convert their face-to-face appointments to telephone or video appointments. In addition, automatic appointment reminder calls and letters to patients were suspended to decrease the likelihood of patients coming into the medical center. Initially, there were not enough medical support assistants/clerical staff to call every patient to convert or schedule their appointments, so their efforts were focused on supporting PC clinics or specific providers in specialty clinics. The rapid transition to telehealth led to a substantial change in providers’ responsibilities, where providers were calling their own patients to convert appointments, providing technical support to their patients for virtual modalities, and developing informal trainings.

New workflows had to be implemented and staff had to be instructed about how to incorporate new modalities. Staff in all 3 clinics began taking on additional roles, with many acting as champions to facilitate the switch to telehealth. One comment was as follows:

Primary care is large, and so we had to have provider champions. We had to have nursing champions. We have MSA champions. And those people are the superusers, I guess. And so, staff would be able to go to them, e-mail them about different questions or issues they were having.

PC respondent #160

We identified many other facilitators to the rapid implementation of telehealth. The primary facilitator was that most providers, particularly in HBPC, had experience providing care over the phone for follow-ups and medication management prior to COVID-19. Further, all providers were required to take VVC training courses prior to the pandemic, and although some reported taking the course again at the onset of the pandemic, most were at least cursorily familiar with the technology. Several of the respondents reported having previous experience with video technologies via consultation appointments with patients at a CBOC through clinical video telehealth. Additionally, some providers had used VVC prior to the pandemic. Although most clinics did not have the support or equipment necessary to widely use VVC, some respondents did report using VVC for “warm handoffs,” whereby a physician would conduct a face-to-face visit with a patient and then connect with another subspecialty physician for consult. In other instances, VVC was used to manage chronic diseases, such as high blood pressure and diabetes.

### Telehealth Scheduling

The successful transition to telehealth appointments was largely dependent on the level of communication between the scheduling clerk and the provider in each clinic. Each clinic had its own scheduling infrastructure, which in turn significantly impacted the way the clinic’s providers perceived the transition to telehealth. In HBPC, where providers always schedule their own patients, scheduling was neither mentioned as a concern nor perceived to be a barrier to conducting a telehealth appointment. In PC clinics, where there exists a close relationship between PC providers and scheduling clerks, respondents reported limited scheduling challenges and confusion, especially since PC telehealth scheduling grids were set up before specialty clinics. Therefore, for PC, scheduling was not described as a major barrier to telehealth adoption, even though, during the first 3 months after the onset of COVID-19, when PC scheduling grids had not been created yet, there were double or triple bookings across multiple modalities (telephone, video, and in-person). In contrast to both HBPC and PC, cardiology providers do not schedule their own patients. They are supported by scheduling clerks, who are not closely integrated into cardiology clinics. Therefore, almost all respondents from the cardiology clinic described scheduling as a key barrier to smooth telehealth adoption. This was due to the following 2 major factors: (1) a delay in the establishment of new telehealth scheduling grids, and (2) communication barriers between cardiology providers and scheduling clerks. The combination of these 2 factors resulted in high levels of confusion and frustration about both how to schedule the different modalities and how to effectively complete the patient encounters. A cardiology provider explained:

There were times that we’ve had 10 patients scheduled or more, because whoever was scheduling didn’t realize there’s a separate face-to-face, phone, and VVC grids, but they’re in parallel…. and without a core group of schedulers … those types of scheduling errors have come up.

Cardiology respondent #114

### Telehealth Modalities

Figures 2-4 also illustrate the monthly numbers of telephone and VVC visits in PC, cardiology, and HBPC 12 months before (March 1, 2019, through February 28, 2020) and 12 months after the onset of COVID-19 (March 1, 2020, through March 1, 2021) at GLA. Before COVID-19, the main telehealth modality was telephone, and there was very little, if any, VVC use. At the onset of COVID-19, for PC and HBPC, there was a decrease in telephone use (more so in HBPC than in PC), while video (or VVC) use started to increase for all 3 clinics. VVC use slightly increased for PC and cardiology after the onset of COVID-19, whereas for HBPC, there was a greater increase in VVC use compared to PC and cardiology.

Supporting our quantitative findings, respondents across all clinics and service roles described a heavy reliance on telephone as the main modality of choice in the initial transition period to telehealth. Providers were encouraged to use video conferencing, where video received more workload credits compared to telephone. The video platforms, however, did not have enough bandwidth during the first couple of months of the pandemic outbreak, and there were many glitches due to the sheer volume of people using them. Providers reported that most patients preferred using the telephone. Many patients did not have the proper equipment (internet, bandwidth, email address, and/or computer, smartphone or tablet) to conduct a video visit or found the technology difficult to navigate. However, within a few months of the pandemic, GLA offered iPads to qualified veterans. One comment was as follows:

They [patients] prefer us calling them. That would be the preferred method, if you asked them. It’s just a lot easier, they’re much more comfortable with that method versus having to deal with connection and the microphone doesn’t work. So, for most of our patients, or my patients, they prefer the telephone.

HBPC respondent #107

In addition, not all clinics were sufficiently resourced to allow for all clinical staff to conduct video visits. Providers were now tasked with rescheduling patients from a face-to-face appointment to a telehealth modality and helping patients navigate the telehealth experience, on top of their normal clinical duties. This new process proved to be challenging. One provider described it taking 10 to 15 minutes of a 30-minute appointment slot to explain to a patient how to get onto a VVC video link.

The VVC platform was described as confusing for both patients and providers. Recognizing this limitation to video adoption, VA adopted alternative video platforms based on federal government wide guidance [24] and approved the use of Doximity and FaceTime as options. VVC could only be used if patients had an email address to which to send the appointment link. Multiple links were sometimes sent, so patients and providers ended up on different appointments. In contrast, video appointment links using Doximity could be sent via text message or providers could simply call iPhone users via FaceTime. Notwithstanding these challenges, when conducting video visits with patients, most providers and schedulers defaulted to first scheduling a VVC encounter. When issues with VVC would arise, providers would switch to either Doximity or FaceTime, as both were described as more user friendly. If none of these options worked, providers would default to a telephone call. Some providers described only using the phone, because they did not have the time to navigate the numerous steps required to successfully conduct a video visit.

Providers described the need to continue including in-person visits in their treatment plans. Some providers started seeing more patients face-to-face starting in June 2020, when GLA reauthorized nonurgent procedures and began expanding services to 25% of what they were before the pandemic began. One respondent noted:

[The patients] want to see me face-to-face. And you know, I also have a need to see them face-to-face. So, there are certain patients now I’m starting to just put them on a face-to-face visit because they are not doing well with their depression and social isolation aspect of it, being home. And a provider aspect of it—I miss my patients. I miss the face-to-face appointments. So, the majority [of visits are] telemedicine, but we are seeing 10-15% of our patients face-to-face now.

Cardiology respondent #103

## Discussion

### Principal Findings

This paper documents the rapid expansion process of telehealth services at a VA medical facility and associated CBOCs in Greater Los Angeles across 3 specialties, namely, PC, cardiology, and HBPC. Prior to COVID-19, virtual care was minimal and limited to mostly telephone visits. At the onset of COVID-19, the use of telephone-based virtual care increased substantially for all 3 clinics. However, video-based care slightly increased at PC and cardiology clinics, but the shift to video-based care was most pronounced for HBPC. The following 3 main themes emerged from the interviews regarding the transition to telehealth services: (1) expansion, (2) scheduling, and (3) modalities. Facilitators to telehealth implementation included staff champions, prior telehealth experience, provider trainings, and patient consultations. Barriers included poor video bandwidth, lack of scheduling and IT support, lack of telehealth scheduling grids, and patient preferences.

The decline in the absolute number of patients accessing outpatient services at all 3 clinics after the onset of the pandemic mirrors the national reduction in health care utilization due to restrictions on face-to-face patient care [25]. The rapid transition to telehealth services provided an essential access point for health care use, as demonstrated by the substantial increase in the use of telehealth services for all 3 clinics during the first 3 months of COVID-19, despite significant barriers to adoption. With the relaxation of pandemic restrictions in August 2020, use of telehealth services started to decline for all 3 clinics. However, it never reached pre–COVID-19 levels during the 12-month post–COVID-19 period. More recent levels of telehealth use may indicate a permanent change in telehealth use.

As would be expected, the rapid expansion to incorporate virtual services had its challenges. Even though telehealth technology has advanced since the onset of the pandemic, additional considerations are needed to better respond to the needs of both providers and patients. Technological issues must be addressed at the forefront of telehealth evolution to achieve access for all patient populations with different socioeconomic backgrounds, living situations and locations (eg, living alone and rural vs urban), and health conditions. Several important factors, such as number of steps required to connect to a virtual visit, flexibility in using different types of video-capable platforms, and provision of free or low-cost infrastructure (including devices and internet access), need to be considered for successful adoption of telehealth. Furthermore, scheduling and staffing considerations, such as clear communication strategies between schedulers and providers, as well as provision of support or technical staff to assist patients on how to use VVC (or other telehealth modalities) can help alleviate pressures on the clinical team. The VA has responded to these challenges with a nationwide directive to incorporate a test call standard operating procedure into VVC workflow to ensure veterans are prepared for their VVC visit. Our study findings suggest that when telehealth is more novel to particular areas, such as the cardiology clinic, additional efforts will likely be needed to ensure a smooth transition. Furthermore, the relative increased use of video-based care at HBPC compared to PC and cardiology might allude to the different team structures, the size and scope, as well as the types of services offered at each clinic. Some HBPC services might be more suited for virtual care as the nature of the care is interdisciplinary, where multiple team members, such as nurses, social workers, physicians, and dietitians, have frequent contacts with patients. Additionally, other facilitators include identifying clinic telehealth champions and developing workflows to better guide the incorporation of telehealth modalities into overall treatment plans.

### Limitations

The study has several limitations. First, the study was conducted at a VA site that serves predominantly urban and suburban veterans, limiting its generalizability to dissimilar VA sites. However, a major strength of the study is that very few studies, if any, have compared the expansion of telehealth across multiple specialties during COVID-19. This study identified key challenges and solutions that were both similar and different among the 3 clinics with regard to telehealth implementation during COVID-19. The study focused on one site, with the goal of identifying key learned lessons that could help create a rapid evidence-based research agenda for future multisite studies. Second, the veterans’ perspectives are not represented in this study. Instead, the study’s main objective was to interview providers and administrative staff to understand how telehealth was implemented at a specific site. Future research would benefit from delving into the patient perspective. This paper does not report on the patient demographics of telehealth use, since this is beyond the scope of the study. Future research should examine the patient characteristics of telehealth use in the context of the 3 different clinics. This will provide a better understanding of how best to optimize telehealth implementation for diverse patient populations. Given that the aim of the study was to explore the patterns of telehealth use and identify the barriers and facilitators of rapid implementation of telehealth during COVID-19, the examinations of how workflow changed, how patients were triaged, and how the nature of care changed during the pandemic were beyond the scope of this study. Future studies should explore these issues.

### Comparison With Prior Work

Research on access to telecare must address the “digital divide,” as select groups, such as older individuals living in rural areas and individuals with socioeconomically disadvantaged backgrounds, may be more vulnerable to having limited access to the internet and/or camera-enabled devices [26,27]. Since 2016, the VA has provided tablets/iPads to qualified veterans in order to begin to address this digital divide. To date, there are over 100,000 devices in the field, and these loaned devices also have helpdesk setup assistance. In a recent VA study, however, 20% of tablet recipients (n=604; mean age 56 years, SE 0.20 years) did not use VA-provided tablets, and 33% who had technological difficulties or multiple comorbidities preferred in-person visits to televisits [28]. Another recent study on older veterans, where 36% lived in rural areas (n=118; mean age 72.6 years, SD 8.3 years) found that having access to tablets/iPads may not solve all of the problems of accessibility or use of telecare services. For instance, availability of an internet connection, especially in rural areas, is still a major barrier. In this study, GLA providers at all 3 clinics were able to request tablets/iPads for qualified veterans during COVID-19, but we did not examine the extent to which these tablets were used.

Almost 1 year after the onset of the COVID-19 pandemic, there are still new lessons to be learned about a variety of important topics in telecare. Telehealth is here to stay, although the extent of its longer-term adoption will vary. Nearly every provider in the study noted that they would like to continue utilizing telehealth modalities as a regular part of their care. Therefore, more research is needed to continue identifying which clinical services are better suited for telecare versus in-person traditional care; which services are better suited for the video modality versus the telephone modality; ways to increase access to virtual care for all patient populations; how to assess quality of telecare for different types of services; and finally, how best to integrate telecare with traditional in-person care.

### Conclusion

The movement to integrate telehealth into clinical practice has been growing for several years, but there have been significant barriers to widespread adoption. The COVID-19 pandemic, however, forced rapid expansion of telehealth services. This study provides an overview of telehealth use before and after the onset of COVID-19 and how telehealth was implemented at PC, HBPC, and cardiology clinics at GLA, with a key focus on the challenges that providers and administrators experienced, and the structures and processes that evolved in response to these challenges. Exploring the adoption of telehealth within a single VAMC has provided the opportunity to understand the varied barriers and facilitators of different clinics and care providers. An individual VAMC is an umbrella for a multitude of clinics and service groups, each with distinct needs and priorities. Our findings highlight the flexibility and creativity of VA clinical staff and leadership to rapidly respond to a massive disruption in health care, which required tailoring care delivery at each of the 3 clinics after the onset of COVID-19. The challenges to this process provide lessons for other types of rapid program implementations. This underscores the need to understand individual clinic processes and workflows, in order to provide appropriate resources for each clinic to expand telehealth services. Further, the VA has the largest telehealth program in the nation and is a leader in the provision of virtual care [29]. Therefore, the accelerated expansion of VA telehealth services during COVID-19 was not surprising. Nonetheless, this rapid implementation of telehealth services provides opportunities to apply lessons learned to other VA facilities and non-VA clinical settings. More importantly, the unprecedented expansion of telehealth during COVID-19 provides opportunities to create advanced telehealth solutions [30] that improve access to care for patients and enhance health care professionals’ abilities to deliver care beyond the period of the pandemic.

## Appendix

Multimedia Appendix 1 Outpatient care at Veterans Affairs Greater Los Angeles, California by clinic type before and after the onset of COVID-19.

## Abbreviations

CBOC: community-based outpatient clinics
CPT: current procedural terminology
GLA: Greater Los Angeles, California
HBPC: home-based primary care
PC: primary care
VA: US Department of Veterans Affairs
VAMC: US Department of Veterans Affairs medical center
VVC: US Department of Veterans Affairs Video Connect
