# Supplementary material for: Implementation of Telehealth Services at the US Department of Veterans Affairs During the COVID-19 Pandemic: Mixed Methods Study
Source: JMIR Form Res. 2021 Sep 23;5(9):e29429. doi: 10.2196/29429 (PMC8462492; doi:10.2196/29429)
Supplement: Multimedia Appendix 1 [file formative_v5i9e29429_app1.docx]

| **Multimedia Appendix 1. Outpatient care at Veterans Affairs Greater Los Angeles, California by clinic type before and after the onset of COVID-19.** | | | | | |
| --- | --- | --- | --- | --- | --- |
|  | **Onset of COVID-19*** | **Number of Visits** | **% Change**  **(visits)** | **Number of Patients** | **% Change**  **(patients)** |
| **Primary Care** | Before | 299,881 | -17.4% | 64,361 | -24.3% |
|  | After | 247,849 |  | 48,729 |  |
| **Cardiology** | Before | 14,229 | -24.1% | 5,527 | -33.2% |
|  | After | 10,800 |  | 3,690 |  |
| **HBPC** | Before | 4,102 | -4.2% | 240 | -32.5% |
|  | After | 3,929 |  | 162 |  |
| * Note: Before: 12-months before onset of COVID-19 (March 1, 2019 thru February 28, 2020)  After: 12-months after onset of COVID-19 (March 1, 2020 thru March 1, 2021) | | | | | |
